# Labeling and sorting of avian primordial germ cells utilizing *Lycopersicon Esculentum* lectin

**DOI:** 10.1111/dgd.12948

**Published:** 2024-11-09

**Authors:** Hiroko Iikawa, Aika Nishina, Mizuki Morita, Yuji Atsuta, Yoshiki Hayashi, Daisuke Saito

**Affiliations:** ^1^ Graduate School of Systems Life Sciences Kyushu University Fukuoka Japan; ^2^ Department of Biology, Faculty of Science Kyushu University Fukuoka Japan

**Keywords:** Avian, lycopersicon esculentum lectin, primordial germ cell, sorting

## Abstract

Avian species are essential resources for human society, with their preservation and utilization heavily dependent on primordial germ cells (PGCs). However, efficient methods for isolating live PGCs from embryos remain elusive in avian species beyond chickens, and even in chickens, existing techniques have shown limited efficiency. In this study, we present a rapid, simple, and cost‐effective method for labeling and sorting circulating‐stage PGCs across various avian species, including Carinatae and Ratitae, using *Lycopersicon Esculentum* (Tomato) lectin (LEL). Notably, this method demonstrates high sorting efficiency by identifying a wide range of PGC subtypes while preserving the proliferative and migratory potential of chicken PGCs. This approach is anticipated to significantly contribute to the conservation, research, and agricultural industries related to avian species globally.

## Graphical abstract

Avian species are essential resources for human society, with their preservation and utilization heavily dependent on primordial germ cells. We present a rapid, simple, and cost‐effective new method for labeling and sorting circulating‐stage primordial germ cells across various avian species. This approach is anticipated to significantly contribute to the conservation, research, and agricultural industries related to avian species globally.

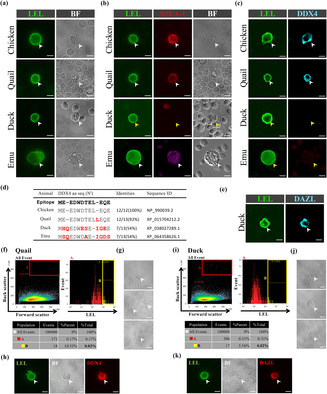

## 1 INTRODUCTION

Avian species play crucial roles in various sectors including industry, agriculture, and scientific research. Consequently, conserving avian biological resources and implementing selective breeding and genetic manipulation are imperative. However, the established methods for cryopreservation of avian fertilized eggs, even in most common species like chickens, remain limited, and sperm cryopreservation techniques are also constrained (Svoradova et al., 2021). Although advancements are being made in avian embryonic stem cells (ESCs) and induced pluripotent stem cells (iPSCs) research (Dai et al., 2014; Pain et al., 1996), challenges persist in utilizing these undifferentiated cells for lineage preservation and the generation of genetically modified birds.

In this context, primordial germ cells (PGCs), the precursors of reproductive cells, have emerged as valuable tools for germline preservation, selective breeding, and the generation of genetically modified birds. During early development, PGCs undergo long‐distance migration to reach the gonads (Richardson & Lehmann, 2010). In avian species, PGCs utilize the circulatory vascular system as their migratory route (Saito et al., 2022; Swift, 1914). The circulating PGCs in birds offer the potential for generating germline chimeras through their collection from the bloodstream and their transplantation into recipient embryo's vasculature (Saito et al., 2022; Tajima et al., 1993). These chimeras, harboring donor germ cells in their reproductive organs have been shown to transmit donor germ cells to subsequent generations (Tajima et al., 1993). Moreover, PGCs are amenable to cryopreservation (Moore et al., 2006). Recent advancements in chicken PGC cultivation techniques have facilitated generating genetically modified PGCs, thereby initiating the production of transgenic chickens through transplantation into embryonic vasculature (Whyte et al., 2015; van de Lavoir et al., 2006).

Despite the significant utility of PGCs, their limited abundance within embryos underscores the critical need for efficient techniques for their collection. However, several challenges must be addressed in collecting PGCs from avian species. For example, in chickens, a common approach involves labeling PGCs derived from bloodstream or gonads with stage‐specific embryonic antigen‐1 (SSEA‐1) antibody, which recognizes specific glycans on the cell membrane of chicken PGCs (Jung et al., 2005), and subsequently isolating them by fluorescence‐activated cell sorting (FACS) (Ichikawa et al., 2022; Mozdziak et al., 2005). This method has several drawbacks, including its time‐consuming procedural complexity, which often leads to sample loss, and the inability to label all PGCs (De Melo Bernardo et al., 2012). Although isolation of PGCs would be more feasible and efficient in reporter transgenic chicken lines specifically labeling PGCs (Chen et al., 2023; Hagihara et al., 2020; Rengaraj et al., 2022), the global availability of such chicken lines is not guaranteed. Furthermore, in avian species other than chickens, the detection of PGCs using SSEA‐1 is more limited. For instance, it has been reported that PGCs in the bloodstream and gonads of Carinatae birds including the Japanese quail, Mallard duck, Muscovy duck, and turkey, as well as in the bloodstream and gonads of Ratitae bird such as ostrich, cannot be stained with SSEA‐1 (D'costa & Petitte, 1999, Jung et al., 2017, Hassanzadeh et al., 2019). Therefore, compared to existing methods, the development of a more versatile, simple, rapid, efficient, universally accessible, and cost‐effective technique would significantly enhance research progress. However, currently, no method meets all these criteria.

In this study, we have addressed these challenges with a comprehensive solution. Our approach involves the utilization of *Lycopersicon Esculentum* (Tomato) lectin (LEL) for the labeling and isolation of avian PGCs. LEL, a protein capable of recognizing and binding to specific sugar chain, the poly‐N‐acetyl D‐glucosamine sugar residues (Nachbar & Oppenheim, 1982), has been employed for this purpose. Our findings demonstrate that LEL selectively accumulates on the cell membrane of viable PGCs across a broad spectrum of avian species, ranging from Carinatae to Ratites. Notably, the precision of PGC recognition achieved with LEL is remarkably high, potentially exceeding that of the SSEA‐1 antigen and aligning with the established PGC markers, DEAD‐box helicase 4 (DDX4) and *deleted in azoospermia‐like* (DAZL). Furthermore, the labeling process is exceedingly rapid, with results obtained within 15 mi, and involves minimal procedural steps, thereby mitigating potential adverse effects on PGCs and minimizing sample loss associated with traditional staining techniques. Moreover, our method exhibits high sorting efficiency in all examined species. Critically, post‐sorting analyses confirm that proliferation efficiency and homing ability of chicken PGCs remain unaffected.

## 2 RESULTS

### 2.1 LEL specifically labels PGCs in E2.5 chicken blood samples

Lectins, which recognize glycan structures on cell surfaces, represent an ideal class of molecules for labeling live cells and sorting them through extracellular applications (Nicola et al., 2005). Their rapid binding may also render lectins more efficient than traditional immunostaining, allowing for faster and simpler results. Specifically, LEL has been shown to react with PGCs in fixed sections from mouse, pig, and chicken embryos (Ojeda & Icardo, 2006; Takagi et al., 1997). Moreover, LEL has been demonstrated to label circulating PGCs in the vasculature of Embryonic day (E2.5) chicken embryos (Saito et al., 2022). Therefore, we initially aimed to verify whether LEL can specifically label chicken PGCs in blood samples from Hamburger and Hamilton's stage (HH) 14–15 (Hamburger & Hamilton, 1951) (E2.5) embryos and assess the pattern and intensity of LEL labeling with that of SSEA‐1, a commonly used marker for membrane labeling and sorting of chicken PGCs (Ichikawa et al., 2022; Karagenc et al., 1996).

**FIGURE 1:**
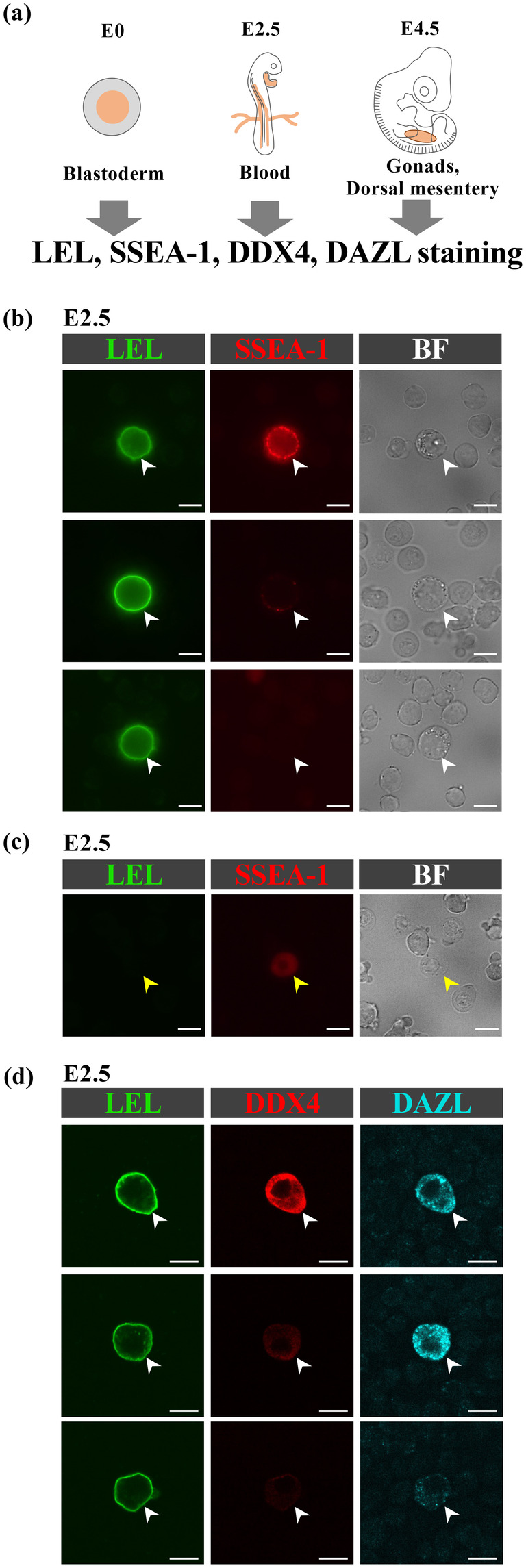
LEL specifically labels PGCs in E2.5 chicken blood samples. (a) Schematic drawing of obtained cells/tissues (orange). (b, c) E2.5 live blood samples stained by LEL and SSEA‐1. (d, e) E2.5 fixed blood samples stained by LEL with DDX4 or DAZL, respectively. (f) The Venn diagram showing the relative abundance of SSEA‐1‐, DDX4‐, and DAZL‐positive cells in LELpositive population, respectively. (g) E0 live blastodermal cells stained by LEL and SSEA‐1. (h) E4.5 live gonadal tissue stained by LEL and SSEA‐1. White and yellow arrowheads indicate PGCs and blood cells, respectively. BF denotes the bright field image. White and yellow scale bars indicate 10 and 200 μm, respectively.

First, regarding the staining pattern and intensity of SSEA‐1: When examining the morphology of cells with SSEA‐1 signal localized on their membranes in the collected blood samples, all cells exhibited PGC‐like characteristics, including a large size (approximately 14 μm in diameter) and distinctive cytoplasmic features such as oil droplets and glycogen granules (Ando & Fujimoto, 1983; Macdonald et al., 2010) (Figure 1a,b). However, the signal intensity varied among cells (Figure 1b). Notably, adjusting the exposure to detect PGCs with the weakest SSEA‐1 signal also led to the detection of nonspecific autofluorescence in blood cells (Figure 1c).

In contrast, in the case of LEL staining, the signal was localized on the cell membranes, and all cells exhibiting the LEL signal consistently displayed PGC‐like morphology. Unlike SSEA‐1 staining, the signal intensity was uniform and robust, showing no variation (Figure 1b). Additionally, the co‐staining of these two signals revealed that all SSEA‐1‐positive cells were included within the LEL‐positive cell population, and the number of LEL‐positive cells was 1.5 times greater than that of SSEA‐1‐positive cells (55.0%; 330 SSEA‐1‐positive cells/600 LEL‐positive cells) (Figure 1b). Given the reported heterogeneity in SSEA‐1 antigen expression in chicken PGCs, indicating incomplete labeling (De Melo Bernardo et al., 2012), LEL staining not only surpasses SSEA‐1 in PGC recognition but also suggests the ability to detect certain subsets of heterogeneous PGCs undetectable by SSEA‐1.

To verify whether the LEL‐positive and SSEA‐1‐negative cells, which exhibit morphological characteristics of PGCs, are indeed PGCs, we performed concurrent staining with DDX4 or DAZL antibody, two of the most common and reliable markers for PGCs (Rengaraj et al., 2022; Tsunekawa et al., 2000). Blood samples were fixed and immune‐stained with DDX4 or DAZL, followed by LEL staining (Figure 1a,d). High‐magnification observation using confocal microscopy revealed that all LEL‐positive cells were indeed positive for DDX4 and DAZL (100%; 127 DDX4 and DAZL double positive cells/127 LEL positive cells), though approximately 10%–20% exhibited very weak expression of these markers (Figure 1d). These findings indicate that LEL can label all PGCs within the E2.5 population. Furthermore, despite variability in DDX4 and DAZL expression levels, LEL consistently and robustly identifies all PGCs.

In the subsequent investigation, we aimed to determine whether LEL could specifically label chicken PGCs at developmental stages other than E2.5. We conducted co‐staining experiments using LEL and SSEA‐1 antibody on samples from Eyal‐Giladi and Kochav's stage X (EGK stX; E0) (Eyal‐Giladi & Kochav, 1976) and HH24‐25 (E4.5) stages, representing pre‐ and post‐PGC migration stages, respectively (Figure 1a). In dissociated samples from E0 blastoderms, SSEA‐1 staining was absent (Figure S1a), suggesting that PGC recognition via SSEA‐1 was not feasible at this stage. However, LEL signal was detected in 70.7% of all examined cells (769 LEL‐positive cells/1088 total cells) (Figure S1a). Given the reported low number of PGCs at E0 (Tsunekawa et al., 2000), it is unlikely that LEL staining is PGC‐specific at E0. In dissociated samples from E4.5 chicken gonads and dorsal mesentery of 15 embryos, LEL‐positive cells were found to be 6.3 times more abundant than SSEA‐1‐positive cells. All SSEA‐1‐positive cells were also LEL‐positive, and most exhibited PGC morphology (Figure S1b). However, SSEA‐1‐negative/LEL‐positive cells (88.4%; 722 SSEA‐1‐negative cells/817 LEL‐positive cells) did not display PGC morphology (Figure S1b). Therefore, in the gonadal and dorsal mesenteric regions of E4.5 chicken embryos, LEL also reacts with somatic cells, thereby demonstrating low specificity for PGCs at this stage.

Overall, these findings suggest that while LEL is effective in specifically labeling PGCs at E2.5, its specificity decreases at earlier (E0) and later (E4.5) stages, where it appears to bind to a broader range of cell types.

### 2.2 LEL staining is effective for sorting chicken PGC without compromising their proliferation and homing potential

To date, the sorting of live chicken PGCs through fluorescence‐activated cell sorting (FACS) has predominantly relied on techniques employing SSEA‐1 staining (Ichikawa et al., 2022; Mozdziak et al., 2005), except in cases where PGC reporter transgenic chicken lines were utilized. However, as indicated by the results in Figure 1 and previous report (De Melo Bernardo et al., 2012), not all chicken PGCs at E2.5 can be efficiently stained using SSEA‐1 (Figure 1a–c). Given the quicker and simpler process of LEL staining, along with its higher efficiency in PGC staining, it was anticipated to be a valuable tool for sorting circulating PGCs.

**FIGURE 2:**
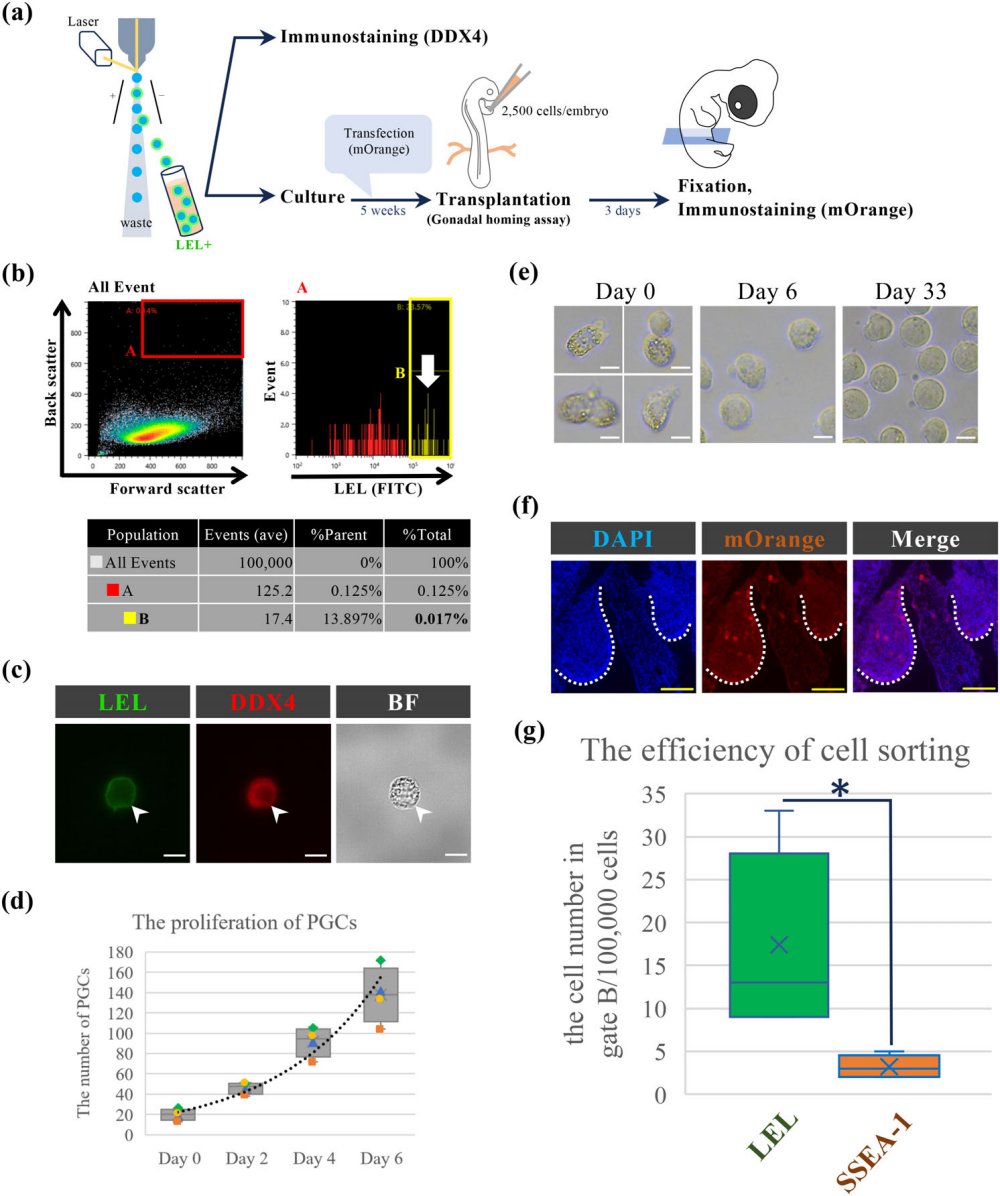
LEL staining is effective for sorting chicken PGC without compromising their integrity. (a) Diagram of experimental flow: E2.5 live chicken blood was stained with LEL, followed by sorting for DDX4 immunostaining, culture, transplantation, and homing assays. (b) Flow cytometry analysis of LEL‐stained E2.5 chicken blood. The dot plots of show forward scattering (FSC; x‐axis) versus back scattering (BSC; y‐axis), with a histogram of FITC fluorescence. The cells in gate B (yellow square) were sorted. A white arrow indicates the peak in gate B. (c) Immunostaining of sorted cells with DDX4. White arrowheads indicate cells stained with DDX4. (d) The proliferation of PGCs from sorted day (Day 0) to 1 week after (Day 6) (*n* = 4). Box plots represent the mean, upper and lower interquartile. (e) Cultured PGCs from the day of sorting (Day 0) to 1 month after (Day 33). (f) Images of transverse section of an embryo injected with LEL‐sorted PGCs at E5.5 (*n* = 3). The white dotted line delineates the gonads area. White and yellow scale bars indicate 10 and 100 μm, respectively.

To verify the feasibility of using LEL staining for sorting PGCs, we collected blood from 10 E2.5 chicken embryos, stained it with LEL, and performed sorting using a cell sorter (Figure 2a). Initially, we gated based on cell size and complexity to exclude most blood cells, followed by a second gate to sort cells with strong DyLight® 488 fluorescence (Figure 2b; Figure S2a). As a result, we isolated a total of 370 LEL‐positive cells from 10 embryos (an average of 37 cells per embryo) (Figure 2b). The sorting efficiency was 0.0174% per 100,000 cells (17.4 ± 10.43 cells per 100,000 cells) (Figure S2a). Examination of sorted cells under bright‐field microscopy confirmed that all cells exhibited the characteristics typical of large, lipid‐rich cells, consistent with PGCs. Further immunostaining with DDX4 antibody revealed that 100% of the sorted cells were DDX4 positive (27/27, 214/214, 228/228, *n* = 3), underscoring the high effectiveness of LEL staining for PGC sorting (Figure 2a,c,g).

To determine if the labeling and sorting process affects the functional properties of PGCs, we analyzed the proliferation ability of the sorted LEL‐positive cells. The sorted PGCs proliferated at a doubling time of 2 days immediately after sorting (Figure 2d), which increased to a doubling time of 1–1.5 days after a week. Initially, a high proportion of PGCs formed cell processes and contained abundant lipid droplets in their cytoplasm (Figure 2e). Over time in culture, the morphology shifted to a more spherical shape, and the number of lipid droplets decreased (Figure 2e). These changes in PGC morphology and cytoplasmic composition are consistent with those observed in PGCs cultured in authentic medium over extended periods (Dehdilani et al., 2023). After 1 month in culture, the morphology of the PGCs resembled that of typical cultured PGCs. These data indicate that LEL staining for PGC sorting does not impede PGC proliferation.

Next, we assessed the homing ability of sorted LEL‐positive PGCs by transplanting them into the bloodstream of recipient chicken embryos at E2.5 (Figure 2a). Post‐incubation, the embryos were examined for the presence of donor‐derived PGCs in the gonads. A significant number of LEL‐positive PGCs successfully homed to the gonads of recipient embryos (Figure 2a,f), demonstrating that the sorting process did not impair the homing ability of the PGCs.

Finally, we investigated whether LEL‐based sorting of chicken PGCs is more efficient than sorting using SSEA‐1 antibodies. By determining the minimum fluorescence intensity required to collect SSEA‐1labeled PGCs with 100% accuracy, we found that the sorting efficiency was 0.0032% (3.2 ± 1.30 cells per 100,000 cells) (Figure S2b,c). According to our method, LEL‐based sorting method was approximately 5.4 times more efficient than the SSEA‐1‐based approach for isolating chicken PGCs from blood samples (Figure 2g).

### 2.3 LEL staining is also effective for labeling and sorting non‐chicken avian PGCs

Reports of successfully isolating PGCs in avian species other than chickens have been scarce. This scarcity primarily arises from the inefficiency of SSEA‐1 in identifying PGCs in non‐chicken avian species and the lack of alternative techniques. Therefore, we sought to validate whether LEL could selectively recognize circulating PGCs in non‐chicken avian species, specifically focusing on Japanese quail, duck (Carinatae), and emu (Ratites).

**FIGURE 3:**
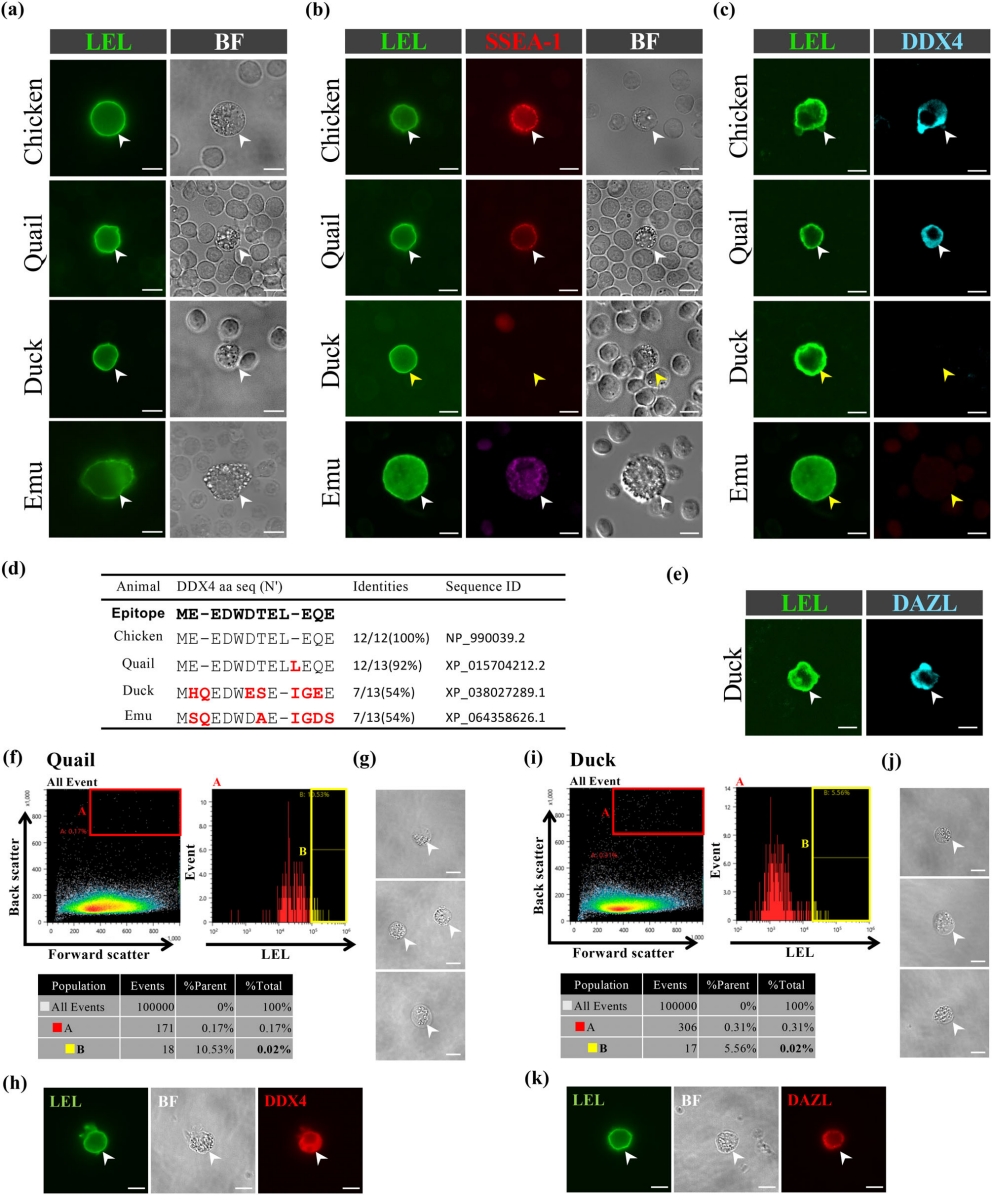
Blood PGCs from other avian species can be stained by LEL and purified by FACS. (a) LEL staining of live blood samples from quail, duck, and emu. (b) SSEA‐1 immunostaining with LEL staining of HH14‐15 live blood samples from chicken, quail, duck, and fixed blood sample from emu. Arrowheads indicate SSEA‐1 stained PGCs (white) and unstained PGCs (yellow). (c) DDX4 immunostaining with LEL staining of fixed HH14‐15 blood from chicken, quail, duck, and emu. Arrowheads indicate DDX4 stained PGCs (white) and unstained PGCs (yellow). (d) Homologous sequences of amino acids in avian DDX4 against the epitope of DDX4 antibody. Red letters indicate non‐conserved amino acids. Homologous sequences were determined by the amino acid alignment using ClustalW (https://www.genome.jp/tools‐bin/clustalw). (e) DAZL immunostaining with LEL staining of fixed HH14‐15 duck blood. White arrowheads indicate DAZL stained PGC. (f, i) Isolation of quail and duck PGCs from HH14‐15 live blood samples by FACS. The cells in the gate B (yellow box) were collected. (g, h, j, k) Bright field images of sorted HH14‐15 quail (g) and duck (j) live PGCs. DDX4 (h) and DAZL immunostaining (k) with LEL staining of sorted quail and duck PGCs, respectively. White arrowheads indicate PGCs. Scale bars: 10 μm.

Live blood samples were obtained from embryos of these avian species at stages equivalent to HH14‐15, followed by LEL staining (Figure 3a). In all samples, the presence of LEL‐positive cells was consistently confirmed. Bright‐field examination revealed that these LEL‐positive cells exhibited morphological features characteristic of PGCs, with no LEL signal detected in other blood cells (Figure 3a). Comparison with SSEA‐1 revealed that only a small subset of LEL‐positive live cells in Japanese quail were SSEA‐1 positive (40.7%; 176 SSEA‐1‐positive cells/432 LEL‐positive cells), and no SSEA‐1 staining was detected in duck live PGCs, while they were positively stained by LEL (0%; 0 SSEA‐1‐positive cell/100 LEL‐positive cells) (Figure 3b). In the case of the emu, although live samples were not tested, fixed samples showed that only 38.5% of LEL‐positive cells were SSEA‐1 positive (5 SSEA‐1‐positive cells/13 LEL‐positive cells) (Figure 3b). Further comparison between DDX4 and LEL signals showed that in quail, a large subset of LEL‐positive cells was DDX4 positive (96.1%; 98 DDX4‐positive cells/102 LEL‐positive cells), similar to chicken PGC staining (Figure 3c compared to Figure 1d). However, no DDX4‐positive cells were found in duck and emu samples (0% in duck; 0 DDX4‐positiv cell/70 LEL‐positive cells, 0% in emu; 0 DDX4‐positive cell/13 LEL‐positive cells) (Figure 3c). This limited DDX4 staining in duck and emu PGCs may result from low cross‐reactivity with antibodies generated against chicken DDX4, due to the poor conservation of the N‐terminal amino acids sequence, which is the epitope for the DDX4 antibody, among species (Figure 3d). In duck samples, 98.3% of LEL‐positive cells were DAZL positive (172 DAZL‐positive cells/175 LEL‐positive cells) (Figure 3e). Due to a shortage of samples, DAZL staining were not conducted for emu. Altogether, LEL exhibited high specificity in labeling circulating PGCs in Japanese quail, duck, and emu.

Finally, we explored the feasibility of sorting LEL‐positive PGCs using a cell sorter for Japanese quail and duck. Blood was collected from HH14‐15 quail or HH14‐15 duck embryos, stained with LEL, and sorted using a cell sorter. Cells were gated based on strong DyLight® 488 fluorescence, as in the chicken experiments (Figure 3f,i). We isolated a total of 325 LEL‐positive cells from 11 quail samples (29.5 PGCs/embryo) with a 0.02% sorting efficiency (18 cells per 100,000 cells) (Figure 3f), and a total of 1351 LEL‐positive cells from 15 duck samples (90.1 PGCs/embryo) with a 0.02% of sorting efficiency (17 cells per 100,000 cells) (Figure 3i). Examination under bright‐field microscopy confirmed that all sorted cells exhibited the typical morphology of PGCs in both quail and duck samples (Figure 3g,j). The sorted LEL‐positive quail and duck cells were confirmed to be DDX4‐positive (100%, 8/8, 62/62, 74/74, 49/49, *n* = 4) and DAZL‐positive (96.8%, 120 DAZL‐positive cells/124 LEL‐positive cells), respectively (Figure 3h, k). These results demonstrate that LEL‐based PGC sorting is applicable not only to chickens but also to quail and duck.

## 3 MATERIALS AND METHODS

### 3.1 Animals

Fertilized eggs from the BL‐E strain of brawn leghorn chickens and the WE strain of Japanese quail were provided by the Nagoya University through the National Bio‐Resource Project of the MEXT, Japan. Additional fertilized chicken eggs were sourced from Yamagishi (Mie, Japan). Fertilized duck and emu eggs were acquired from Takahashi jinko fukajo (Osaka, Japan) and Kiyama farm (Saga, Japan), respectively. Chicken, Japanese quail, and duck eggs were incubated at 38.5°C, while emu eggs were incubated at 36.0°C in a high‐humidity chamber (50%–60%). Chicken embryos at stage E0, E2.5, and E4.5 were dissected and collected after 30 min, 60 h, and 72 h of incubation, respectively. Blood samples from quail, duck, and emu embryos were collected after 53, 86, and 144 h of incubation, respectively. All animal experiments were adhered to the ethical guidelines for animal experimentation established by Kyushu University.

### 3.2 Sample collection and preparation for staining

Blastoderms, blood, and gonads (including dorsal mesentery tissue) were collected from EGK X (E0), HH14‐15 (E2.5), and HH24‐25 (E4.5) embryos (Eyal‐Giladi & Kochav, 1976; Hamburger & Hamilton, 1951). Samples were placed in PBS (137.93 mM NaCl, 8.10 mM Na_2_HPO_4_, 2.67 mM KCl, 1.47 mM KH_2_PO_4_) on ice prior to the staining. Blastoderms and gonadal tissues were minced into small pieces (approximately 0.3 mm in diameter) and treated with 0.05% trypsin/EDTA/PBS at 37.0°C for 10 min to facilitate dispersion, followed by washing in PBS. Cells were dispersed by pipetting every 3 min, and the trypsinization was limited to 10 min to preserve glycans on PGCs. The reaction was stopped by adding an equal volume of FBS, and cells were centrifuged at approximately 138 g for 5 min. Prior to staining, cells were washed and resuspended in PBS containing 0.2 mg/mL of heparin (PBSh) (Sigma‐Aldrich).

### 3.3 LEL labeling

LEL DyLight® 488 (Vector Laboratories, DL‐1174) was centrifuged at 15,000 rpm, 4°C for 30 min, and the supernatant was carefully collected to remove debris before use. Staining processes were performed on ice or at 4°C in the dark. Blood, dispersed blastoderm, and gonadal cells were incubated with a 1:250 dilution of LEL in PBSh for 5 min. The samples were then washed with 10 times the volume of cold PBSh and centrifuged at 1500 rpm for 5 min. The supernatant was carefully removed, and the washing process was repeated twice.

### 3.4 Co‐staining with SSEA‐1, DDX4, DAZL

For co‐staining with SSEA‐1, cells were processed without fixation, and all steps were conducted on ice or 4°C. After sample collection, the cells were washed with PBS and incubated with a 1:100 dilution of SSEA‐1 (sc‐2170; Santa Cruz Biotechnology) in 5% FBS PBS for 1 h in the dark. Following three washes with PBS, the cells were incubated with donkey anti‐mouse IgM and donkey anti‐mouse IgG conjugated with Alexa fluor 568 (invitrogen) at a 1:200 dilution in 5% FBS PBS for 30 min in the dark. Finally, the cells were washed three times with PBS.

For co‐staining that includes DDX4 or DAZL (LEL/DDX4, LEL/DAZL, LEL/SSEA‐1/DDX4, LEL/SSEA‐1/DAZL), cells were first fixed with 4% paraformaldehyde (PFA) in PBS for 30 min at room temperature (RT) in the dark immediately after sample collection. The cells were then washed twice with TNT buffer (0.1 M Tris, 0.15 M NaCl, and 5 mL Tween20), and incubated in 1% blocking reagent TNT for 30 min in the dark. Subsequently, the cells were incubated with primary antibodies at the following dilutions: 1:100 for SSEA‐1 mouse IgM antibody, 1:500 for anti‐DDX4 rat IgG antibody(Atsuta et al., 2022), or a 1:500 for anti‐DAZL rabbit antibody (ab215718; Abcam). This was done in 1% blocking reagent TNT for 1 h on ice. After washing with TNT, the cells were incubated with secondary antibodies at a 1:200 dilution in 1% blocking reagent TNT for 30 min on ice. The secondary antibodies used were donkey anti‐mouse IgM, donkey anti‐rat IgG, or donkey anti‐rabbit IgG conjugated with Alexa fluor 568 or 647 (invitrogen), depending on the primary antibody.

Finally, LEL was added to the staining samples at a 1:250 dilution, and the samples were incubated for an additional 5 min. The samples were then washed three times with TNT.

### 3.5 Image capturing for cell samples

The stained cell samples were mounted on 96‐well glass‐bottom dishes (AGC techno glass) for observation. For single DyLight® 488 and dual‐color of DyLight® 488 and Alexa568 detection, images were captured using an inverted microscope IX83 (Olympus) equipped with an sCMOS camera, Zyla‐4.2 plus (ANDOR), and operated with microscope software cellSens (Olympus). The acquired images were further processed and edited using Fiji‐ImageJ (NIH).

For capturing two or three‐color images including Alexa647, an inverted microscope IX83 (Olympus) coupled with spinning‐disk confocal microscopy (Dragonfly 200, OXFORD Instruments) was used. The system was equipped with an sCMOS camera, Zyla‐4.2 plus, and a 20x dry objective lens (UPlanSApo, Olympus) or a 40x dry objective lens (UPlanSApo, Olympus). The images were captured and processed using Fusion software 2.3.0.44 (ANDOR) and then edited using Fiji‐ImageJ.

For the emu samples with three‐color staining (DyLight® 488/Alexa568/Alexa647), images were captured using an upright microscope DM6000B (Leica) equipped with an ORCA‐Flash4.0 camera (Hamamatsu) and a 40x dry objective lrns (HCX PL Fluotar, Leica), with the imaging software LasX (Leica).

### 3.6 Cell sorting

LEL‐positive cells were isolated using the SH800 (SONY) fluorescence‐activated cell sorter (FACS). The initial sorting parameters were automatically regulated by the SH800 program, with a typical setup including a Droplet Clock set to 30,500 Hz, Droplet Drive to 19.01, Sort Delay to 30, Sort Phase to 0 degrees, Charge to 50.0, Deflection Left to −638, Deflection Right to 788. Chip alignment and calibration were performed using the manufacturer's fluorescent beads (Automatic Setup Beads: SONY). The cell sorter parameters were fine‐tuned to detect all particles in the sample with low forward scatter (FSC) threshold, specifically setting the threshold at 5.0%. Sensor Gain settings were adjusted to optimize LEL staining detection, with FSC set at 4, back (side) scatter (BSC) at 28%, and FL2 FITC at 30%. Throughout the sorting process, the sample and collection areas were maintained at 4°C. The sample cells were detected by their FSC and BSC, along with their fluorescent signal, representing cell size, complexity, and the presence of LEL staining, respectively. Gate B (histogram of FITC fluorescence) was set so that all sorted cells were morphologically PGCs. Gate A (density dot plot of FSC versus BSC) was set to ensure that all sorted cells in Gate B were plotted within the designated area. The same Gate A was applied for all animal samples. SSEA‐1‐positive cells were isolated using the SH800 in the same manner, with Gate A from LEL sorting also used for SSEA‐1 sorting. For chicken samples, Gate B was set above 10^4^.

### 3.7 Chicken PGC culture

Post‐sorting, chicken PGCs were cultured in FAcs medium in non‐treated 96‐well flat bottom plates (Thermo) following the protocol described by Chen et al. and Saito et al., with modifications based on the original culture method by Whyte et al. (Chen et al., 2019; Saito et al., 2022; Whyte et al., 2015). The FAcs medium composition includes 65.5% DMEM (high Glucose, Ca^2+^‐free, no glutamine) (Nakarai tesque), 21.8% DDW (Fuji film), 1 × Nucleotides (Millipore), 2.0 mM GlutaMax (Gibco), 1.2 mM Soldium Pyruvate (Gibco), 1 × NEAA (Gibco), 55 μM b‐mercaptoethanol (Wako), 1% Penicillin–Streptomycin‐Amphotericin (PSA) (Fuji film), 2% B27 Supplements (Gibco), 0.20% Chicken serum (CS) (Biowest), 0.1 mg/mL Heparin (Sigma‐Aldrich), 2 mg/mL Ovoalbmin (Sigma‐Aldrich), 25 ng/mL Activin A (API), 4 ng/mL FGF2 (Wako), 100 μM CaCl₂ (Nakarai tesque). The PGCs were incubated at 38.0°C with 5% CO_2_ in a humidity incubator. Half of the medium was replaced with fresh medium every 2 days.

### 3.8 Plasmid construction

The pT2A‐CAGGS‐Gap‐mOrange‐IRES2‐PuroR plasmid was constructed by PCR amplification of the DNA encoding the ORF of Gap‐mOrange, IRES2, and the ORF of puromycin resistant gene (PuroR) from RCASBP‐Gap‐mOrange (Murai et al., 2015), pIRES2‐EGFP (Clontech), and pT2A‐CAGGS‐Tet3G‐2A‐PuroR (Saito et al., 2022), respectively. The amplified products were inserted into the NotI‐MluI, XhoI‐EcoRI, and EcoRI‐BglII sites of pT2A‐CAGGS(Tadokoro et al., 2016), respectively.

### 3.9 Gonadal homing assay

After sorting, PGCs were cultured for approximately 5 weeks to allow for proliferation. The cells were then transfected with pT2A‐CAGGS‐Gap‐mOrange‐IRES2‐PuroR and pCAGGS‐T2TP (Sato et al., 2007) plasmids using Lipofectamine 2000 (invitrogen). Successfully transfected cells were selected with puromycin. The selected transfected PGCs were resuspended in OptiMEM (gibco) at a concentration of 2500 cells/μL and injected into HH14‐15 stage host chicken embryos. The embryos were subsequently incubated for 3 days.

Recipient embryos were fixed with 4% PFA/PBS, dehydrated, and embedded in Tissue‐Tek O.C.T. Compound (Sakura Finetek USA), then stored at −80°C. The frozen blocks were sectioned at 14 μm thickness using a cryostat (Thermo scientific) set at −12°C. The sections were placed on grass slides (Matsunami Glass).

The detection of injected PGCs in these sections was performed through immunostaining. The slides were washed with TNT buffer for 5 min, 3 times, and then blocked with 1% Blocking Reagent with TNT for 1 h at RT. The slides were then incubated with a 1:200 dilution of anti‐mCherry (mouse) antibody (Clontech) in 1% Blocking Reagent in TNT at 4°C for 16 h. After this incubation, the slides were washed 3 times with TNT and incubated with a 1:500 dilution of anti‐mouse 568 (goat) antibody (Alexa fluor 568, invitrogen) for 2 h at RT. Following two additional washes, the sections were mounted with cover glass (Matsunami Glass).

For quantification, the gonads from the embryos were dissected, embedded, and gently crushed between glass slides and cover glasses. The number of the injected cell in the gonads was determined using the MVX10 microscope (Olympus).

## 4 DISCUSSION

In this study, we introduced a novel method for labeling avian circulating PGCs utilizing LEL staining. LEL rapidly binds to the cell surface of live PGCs through a simple procedure, demonstrating superior specificity in detecting a larger population of chicken PGCs compared to the conventional SSEA‐1 labeling method. Importantly, our study revealed LEL's capability to recognize PGCs across various avian species beyond chickens. The stained PGCs were effectively sorted using FACS, enabling precise PGC collection with remarkable accuracy. We demonstrated that the labeling and sorting process had no adverse effects on the proliferation and homing ability to the gonads of chicken PGCs.

### 4.1 The heterogeneity of E2.5 chicken PGCs and the recognition specificity of LEL

Previous single‐cell RNA sequencing analyses of E2.5 Dazl::EGFP transgenic chicken PGCs identified a distinct subset characterized by low expression of DDX4 mRNA within the EGFP (DAZL)‐positive cell population (Rengaraj et al., 2022). Additionally, co‐immunostaining of DDX4 and SSEA‐1 revealed a subset with diminished SSEA‐1 antigen expression among DDX4‐positive cells (De Melo Bernardo et al., 2012). These observations underscore the heterogeneity in SSEA‐1 antigen and DDX4 mRNA expression levels within the E2.5 chicken PGC population. Consistent with these findings, our study also detected heterogeneity in the expression levels of DAZL. The observed heterogeneity suggests the existence of multiple subpopulations within the E2.5 PGC population, each potentially possessing distinct characteristics. Alternatively, it may reflect the unstable and fluctuating state of cells during early developmental stages. The precise underlying mechanisms, however, remain to be elucidated. Notably, the glycan recognized by LEL on E2.5 PGCs did not exhibit significant heterogeneity and was capable of identifying PGCs, including those with negative or weak expression of other PGC markers. The discovery of LEL's ability to identify chicken PGCs holds promise for advancing research into PGC heterogeneity.

Our analysis demonstrated that all LEL‐positive cells in the E2.5 chicken blood sample exhibited morphological features consistent with PGCs. Additionally, these cells were more prevalent or exhibited stronger signals compared to those positive for SSEA‐1, DDX4, or DAZL (Figure 1). These results suggest that LEL effectively identifies a broad spectrum of E2.5 PGCs, including those with absent or low expression of SSEA‐1, DDX4, and DAZL. Notably, we achieved a chicken PGC sorting efficiency approximately 3.3 to 5.4 times higher (37 PGCs/sample, 0.0174%) than that previously reported for SSEA‐1‐based sorting (8.45 to 11.1 cells/sample) (Ichikawa et al., 2022) and in this study (0.0032%) (Figure 2b). This enhanced PGC sorting efficiency can be attributed to the broad, uniform, and robust recognition capability of LEL.

### 4.2 The universality of LEL binding to PGC surfaces

This study also highlights the potential of LEL for labeling and sorting PGCs in economically important avian species other than chickens, including quail, duck, and emu. Given the current lack of established methods for specifically labeling live PGCs on the cell surface in these avian species, the ability to sort these cells using LEL represents a significant advancement.

In samples from developmental stages equivalent to HH14‐15 quail, duck and emu, all cells labeled by LEL exhibited morphology characteristic of PGC. Moreover, it is evident that LEL more broadly recognizes PGCs in these avian species than other known PGC markers, as demonstrated by data from chickens. This finding suggests that the glycan targeted by LEL on PGC membranes is evolutionarily conserved across a wide range of avian species, including ancient Ratites and Carinatae birds. The evolutionary conservation of this glycan on PGCs warrants further investigation, as it could also be applied to the conservation of endangered species. If these glycans are associated with PGC migration via the bloodstream, they may also be present in the PGCs of Neoaves and reptiles that exhibit similar migratory behaviors (Golkar‐Narenji et al., 2023).

## AUTHOR CONTRIBUTIONS

H. I. and D. S. designed the study. H. I. and A. N. performed most experiments and analyzed data. M. M. performed a part of the cell sorting experiments. D. S., Y. H. and Y. A. supervised the study. D. S. wrote the manuscript.

## CONFLICT OF INTEREST

The authors declare no competing interests.

## Supporting information


**Data S1.** Supporting information.
