# Supplementary material for: Labeling and sorting of avian primordial germ cells utilizing Lycopersicon Esculentum lectin
Source: Dev Growth Differ. 2024 Nov 9;66(9):452–61. doi: 10.1111/dgd.12948 (PMC11659090; doi:10.1111/dgd.12948)
Supplement: Supplementary file 1 — Data S1. Supporting information. [file DGD-66-452-s001.pdf]

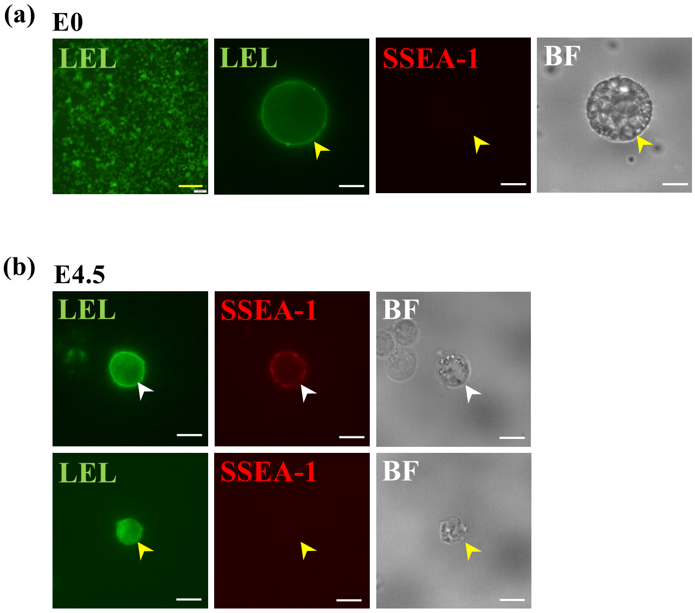

FIGURE S1 Iikawa *et al.*

FIGURE S1 LEL is not specific for chicken PGC at E0 and E4.5 tissues. (**a**) E0 live blastodermal cells stained by LEL and SSEA-1. (**b**) E4.5 live gonadal tissue stained by LEL and SSEA-1. White and yellow arrowheads indicate PGCs and somatic cells, respectively. BF denotes the bright field image. White and yellow scale bars indicate 10 µm and 200 µm, respectively.

(a)

Staining	All	Gate A	Gate B
LEL	100,000	140	33
LEL	100,000	151	9
LEL	100,000	89	13
LEL	100,000	190	23
LEL	100,000	56	9
Average	100,000	125.2	17.4

(b)

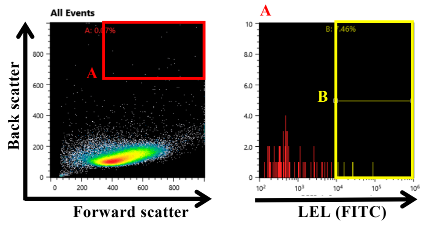

Population	Events (ave)	%Parent	%Total
All Events	100,000	0%	100%
A	104.4	0.104%	0.104%
B	3.2	3.065%	**0.003%**

(c)

Staining	All	Gate A	Gate B
SSEA-1	100,000	67	5
SSEA-1	100,000	158	3
SSEA-1	100,000	103	4
SSEA-1	100,000	75	2
SSEA-1	100,000	119	2
Average	100,000	104.4	3.2

FIGURE S2 Iikawa *et al.*

FIGURE S2 Flow cytometry analysis of LEL or SSEA-1-stained E2.5 chicken blood. (**a**) The cell count data from 5 independent LEL sorting experiments. (**b**) Flow cytometry analysis of SSEA-1-stained E2.5 chicken blood. The dot plots display forward scattering (FSC; x-axis) versus back scattering (BSC; y-axis), with a histogram showing FITC fluorescence intensity. Cells with gate B (yellow square) were sorted. The table below presents the average cell count from 5 independent sorting experiments. (**c**) The cell count data from 5 independent SSEA-1 sorting experiments.
